# Parental recovered acute kidney injury causes prenatal renal dysfunction and fetal growth restriction with sexually dimorphic implications for adult offspring

**DOI:** 10.3389/fphys.2024.1357932

**Published:** 2024-04-12

**Authors:** Jessica F. Hebert, Yoshio Funahashi, Jacqueline M. Emathinger, Megan N. Nickerson, Tahnee Groat, Nicole K. Andeen, Susan B. Gurley, Michael P. Hutchens

**Affiliations:** ^1^ Department of Anesthesiology and Perioperative Medicine, Oregon Health and Science University, Portland, OR, United States; ^2^ Division of Nephrology, Oregon Health and Science University, Portland, OR, United States; ^3^ Operative Care Division, Portland Veterans Administration Medical Center, Portland, OR, United States; ^4^ Department of Pathology, Oregon Health and Science University, Portland, OR, United States; ^5^ Division of Nephrology and Hypertension, Department of Medicine, Keck School Medicine of University of Southern California, Los Angeles, CA, United States

**Keywords:** acute kidney injury, pregnancy, developmental programming, renal function, rhabdomyolysis

## Abstract

**Introduction:** Acute kidney injury (AKI) is rapidly increasing in global incidence and a healthcare burden. Prior maternal AKI diagnosis correlates with later pregnancy complications. As pregnancy influences developmental programming, we hypothesized that recovered parental AKI results in poor pregnancy outcomes, impaired fetal growth, and adult offspring disease.

**Methods:** Using a well-characterized model of rhabdomyolysis-induced acute kidney injury (RIAKI), a form of AKI commonly observed in young people, we confirmed functional renal recovery by assessing glomerular filtration rate (GFR) 2 weeks following RIAKI. We bred sham and recovered RIAKI sires and dams in timed, matched matings for gestational day (GD) 16.5 and offspring (birth–12 weeks, 6 months) study.

**Results:** Despite a normal GFR pre-pregnancy, recovered RIAKI dams at GD16.5 had impaired renal function, resulting in reduced fetoplacental ratios and offspring survival. Pregnant RIAKI dams also had albuminuria and less renal megalin in the proximal tubule brush border than shams, with renal subcapsular fibrosis and higher diastolic blood pressure. Growth-restricted offspring had a reduced GFR as older adults, with evidence of metabolic inefficiency in male offspring; this correlated with reduced renal AngII levels in female offspring from recovered RIAKI pairings. However, the blood pressures of 6-month-old offspring were unaffected by parental RIAKI.

**Conclusions:** Our mouse model demonstrated a causal relationship among RIAKI, gestational risk, and developmental programming of the adult-onset offspring GFR and metabolic dysregulation despite parental recovery.

## Introduction

Acute kidney injury (AKI) is defined by sudden-onset renal cell injury and functional impairment. AKI is diagnosed in over 13 million people globally each year, but subclinical and, consequently, additional undiagnosed cases are common, especially in otherwise healthy adults (Petejova and Martinek, 2014; Zou et al., 2022). Although the recovery of renal function occurs within days to weeks, AKI has been linked to delayed-onset consequences, including increased risk of chronic kidney disease (CKD), mortality, stroke, dementia, hypertension, and fetal growth restriction, costing the United States healthcare system a total of $90 billion per year (Miyazawa et al., 2002; Kelly, 2003; Deng et al., 2004; Kim et al., 2006; Liu et al., 2008; James et al., 2011; Chawla et al., 2014; Makris and Spanou, 2016; Silver and Chertow, 2017; Hebert et al., 2023). CKD, which may result from AKI, further impairs reproductive function, fertility, and germ cell quality in both sexes (Holley and Schmidt, 2013; Palant et al., 2017; Dumanski and Ahmed, 2019; Lundy and Vij, 2019).

Most investigations into the consequences of AKI have focused on older adults; however, multiple etiologies of AKI primarily affect young and otherwise healthy individuals, including sepsis, certain drugs, COVID-19, and rhabdomyolysis (Abdel-Kader and Palevsky, 2009; Makris and Spanou, 2016; Gameiro et al., 2020; Nadim et al., 2020; Turgut et al., 2023). Because of their younger age and expected years of life, reproductive-age AKI survivors are a large population at risk for late AKI-initiated disease. Rhabdomyolysis-induced acute kidney injury (RIAKI) occurs following muscular injury induced by blunt objects (as in assault, motor vehicle crashes, and earthquakes), blast injury, drug-induced obtundation, or physical overexertion (Elterman et al., 2015; Hummel et al., 2016; Stewart et al., 2016). The damaged muscle releases myoglobin into the bloodstream, which is reabsorbed by the renal proximal tubule by megalin and cubilin after glomerular filtration (Gburek et al., 2003). Upon entry into proximal tubule cells, free iron from myoglobin forms reactive oxygen species and causes cell death. Currently, there is no treatment for RIAKI except fluid resuscitation (Lameire et al., 2008; Kodadek et al., 2022).

As men have an overall higher incidence of AKI, outcomes in women are under-characterized (Schiffl, 2020; Loutradis et al., 2021). However, women with renal disease are at risk for adverse maternal and fetal outcomes (Harville et al., 2019; Suarez et al., 2019; Garovic et al., 2022); specifically, a history of recovered AKI in women who later get pregnant increases the risk for preeclampsia and small-for-gestational-age babies (Tangren et al., 2017; Piccoli et al., 2018; Tangren et al., 2018). The maternal kidney controls the systemic vascular tone and blood pressure through the renin–angiotensin system (RAS), primarily via the balance of vasoconstrictive angiotensin II (AngII) to vasodilating factors, which can also influence the localized placental RAS and fetal RAS driving nephrogenesis (Woods et al., 2001; Woods et al., 2004; Lumbers et al., 2019). In addition to the limited studies on maternal outcomes following recovered AKI, no studies have addressed offspring beyond the neonatal stage or the mechanisms of heritable and long-term disease (Perico et al., 2018). Using a well-characterized mouse model of RIAKI, a form of AKI often observed in people of childbearing age (Wilson et al., 1967; Wei et al., 2011; Matsushita et al., 2021), we hypothesized that recovered parental RIAKI would result in poor pregnancy outcomes, impaired fetal growth, and later-onset offspring disease.

## Methods

### Animals

All procedures described below were approved by the Institutional Animal Care and Use Committee of the Portland Veterans Administration Health Care System (protocol #4514-20). The mice were given *ad libitum* access to 5L0D PicoLab Laboratory Rodent Diet (LabDiet), a balanced nutritional rodent chow, at all points of the experiment, including during urinary collection in metabolic cages.

### Rhabdomyolysis model

RIAKI was performed as previously described by our group and others (Wilson et al., 1967; Wei et al., 2011; Matsushita et al., 2021). Male and female 8–12-week-old C57BL/6 mice were subjected to water deprivation for 4 h prior to the procedure. Rhabdomyolysis was induced under isoflurane anesthesia by an anterior thigh intramuscular injection of 50% glycerol in saline (8 mL/kg, half-dose per side), while sham animals were untreated.

### Glomerular filtration rate

The glomerular filtration rate (GFR; uL/min/100 g body weight) was assessed at 24 h and 2 weeks after rhabdomyolysis as the transcutaneous quantification of fluorescein isothiocyanate (FITC)–sinistrin elimination (Scarfe et al., 2018). A region on the abdominal section of the mouse was depilated, and a fluorescence detector (MediBeacon) was applied. After a 5-min baseline reading, FITC–sinistrin (50 μL, 35 mg/mL) was injected into the retro-orbital venous plexus. Elimination was quantified while the mouse was active and awake for 90 min using MediBeacon software. The GFR was calculated using the half-life of elimination.

### Mating and fertility assessment

Both parents may contribute to fetal and placental development, and paternal metabolic health has been shown to influence pregnancy (Galaviz-Hernandez et al., 2019; Eberle et al., 2020); therefore, the sires and dams were subjected to RIAKI and recovery before breeding. After documenting a return to a normal GFR 2 weeks after rhabdomyolysis, timed sham male/sham female and RIAKI male/RIAKI female pairings were established. Female mice were exposed to male mouse urine for 72 h before breeding to stimulate and synchronize estrus before breeding in single-matched pairs; the formation of a vaginal plug was classified as gestational day (GD) 0.5 (Whitten, 1956; Hebert et al., 2021). The time from pair introduction to birth was considered as the days to conception.

### Perinatal mortality and offspring weights

Dams were visually monitored twice daily. The initial litter size was the number of pups present at birth. Perinatal death was calculated as the percentage of initial litter that died or went missing (and presumed ingested) between birth and weaning. Pups were weaned at 3 weeks old and were housed with sex-matched siblings throughout longitudinal assessments. Weights were measured at one-week intervals from birth to 12 weeks (young adulthood cohort). The older adulthood cohort was also weighed at 6 months.

### Pregnancy and placental efficiency assessments

At GD 16.5 (term GD 19.5), urine was collected from pregnant dams for 24 h in metabolic cages. After the GFR was measured, the dams were euthanized by injecting a lethal dose of tribromoethanol. The maternal kidney, heart, placenta, and fetus were all weighed fresh; maternal blood (plasma), kidneys, heart, and placentas, and fetal liver and kidney were collected. All samples were flash-frozen in tubes dipped in liquid nitrogen except the maternal heart and left maternal kidney, which were fixed overnight in 4% paraformaldehyde for histology. Fetoplacental sufficiency was calculated as the ratio of fetal weight/placental weight (Wilson and Ford, 2001).

### Young and older adult offspring assessments

The GFR was measured in 12-week-old (young adult) and 6-month-old (older adult) offspring of RIAKI and sham pairings, as described above, followed by euthanasia by a lethal injection of tribromoethanol. Plasma and laterally bisected right kidneys were flash-frozen after saline perfusion, with hearts and left kidneys perfusion-fixed via the left ventricular apex with 4% paraformaldehyde and collected for histology. Kidneys, hearts, and body weights were all measured.

### Blood pressure measurements

Systolic and diastolic blood pressure, mean arterial pressure (MAP), and pulse were measured before measuring the GFR in GD-16.5 dams and 6-month-old offspring using a computerized tail-cuff system (Hatteras Systems, MC4000 Blood Pressure Analysis System). The 6-month-old mice were acclimated to the measuring system for 3 days, with recordings obtained at the same time each day; no statistical difference was observed in day-to-day measurements. Each animal underwent a cycle of 10 preliminary and 10 experimental measurements daily.

### Urinalysis

Urine was collected for 24 h in urine collection cups pretreated with a protease inhibitor, starting immediately after an animal’s recovery from experimental anesthesia, and measured. Urine was centrifuged at 1,500 g for 15 min to remove particulates; the supernatant was stored at −80°. The total urine protein was measured using a bicinchoninic acid (BCA) kit (Pierce BCA, 23227, Thermo Fisher). Equal volumes of urine were loaded for urine gel electrophoresis; gels were stained with Coomassie Blue and imaged. Urine albumin (Albuwell M, 1011, Ethos Biosciences) and retinol-binding protein 4 (RBP4; ab202404, Abcam) levels were measured by enzyme-linked immunosorbent assay (ELISA).

### Plasma analysis

After collection, blood was centrifuged at 2,000 g for 15 min to obtain plasma, which was separated and frozen at −80°. The blood urea nitrogen (BUN) level was measured in diluted plasma samples using a colorimetric detection kit (EIABUN, Thermo Fisher).

### Quantification of renal brush border proteins

Flash-frozen renal tissue was digested to isolate the renal proximal tubule brush border membranes, as previously described (Biber et al., 1981). In brief, the homogenized mouse renal cortex was homogenized in an isosmotic medium with ethylene glycol tetraacetic acid (EGTA) and precipitated twice with magnesium chloride and centrifuged to obtain a high yield of brush border membranes. The total protein was quantified using a BCA kit. For megalin detection by Western blot, 3 ug of isolated protein was run on 3%–8% Tris–acetate gels (NuPAGE, Invitrogen) at 170 V for 60 min, transferred to polyvinylidene difluoride (PVDF) membranes, and blocked as previously described (Matsushita et al., 2021). The membranes were exposed to a megalin primary antibody (1:1,000; Thermo Fisher) overnight at 4°, washed, and horseradish peroxidase-conjugated secondary antibody-treated (1:5,000; Thermo Fisher) for 2 h at room temperature before final washes and development with SuperSignal West Dura (Thermo Fisher). Images were captured using the ChemiDoc Imaging System (Bio-Rad). The brush border AngII level was determined by AngII ELISA (S-1133, BMA Biomedicals). ACE2 activity was measured following incubation with intramolecularly quenched synthetic ACE2-specific substrate Mca-APK(Dnp) (AnaSpec), as previously described (Wysocki et al., 2014).

### Statistical analysis

Experimental numbers were determined by *a priori* power analyses (power of 80%) using the sample size calculator designed by Wang and Ji (2020) and supported by R (v4.0, package pwr, pwr.anova.test, and pwr.t.test). Statistical analysis was performed using Prism 10.0 (GraphPad). Two-group comparisons were performed using Student’s t-test, with Welch’s correction as appropriate. Multiple-group comparisons were performed with ANOVA (or two-way ANOVA in the case of before–after comparisons in the same mice), with Tukey’s HSD test as appropriate. Risk ratios were calculated with α = 0.05. Statistical significance was considered to be *p* < 0.05. Figures present all data as dots with means and standard error. Any unmarked comparisons in the figures are considered statistically not significant.

## Results

### Rhabdomyolysis causes recoverable acute kidney injury in male and female mice

**FIGURE 1:**
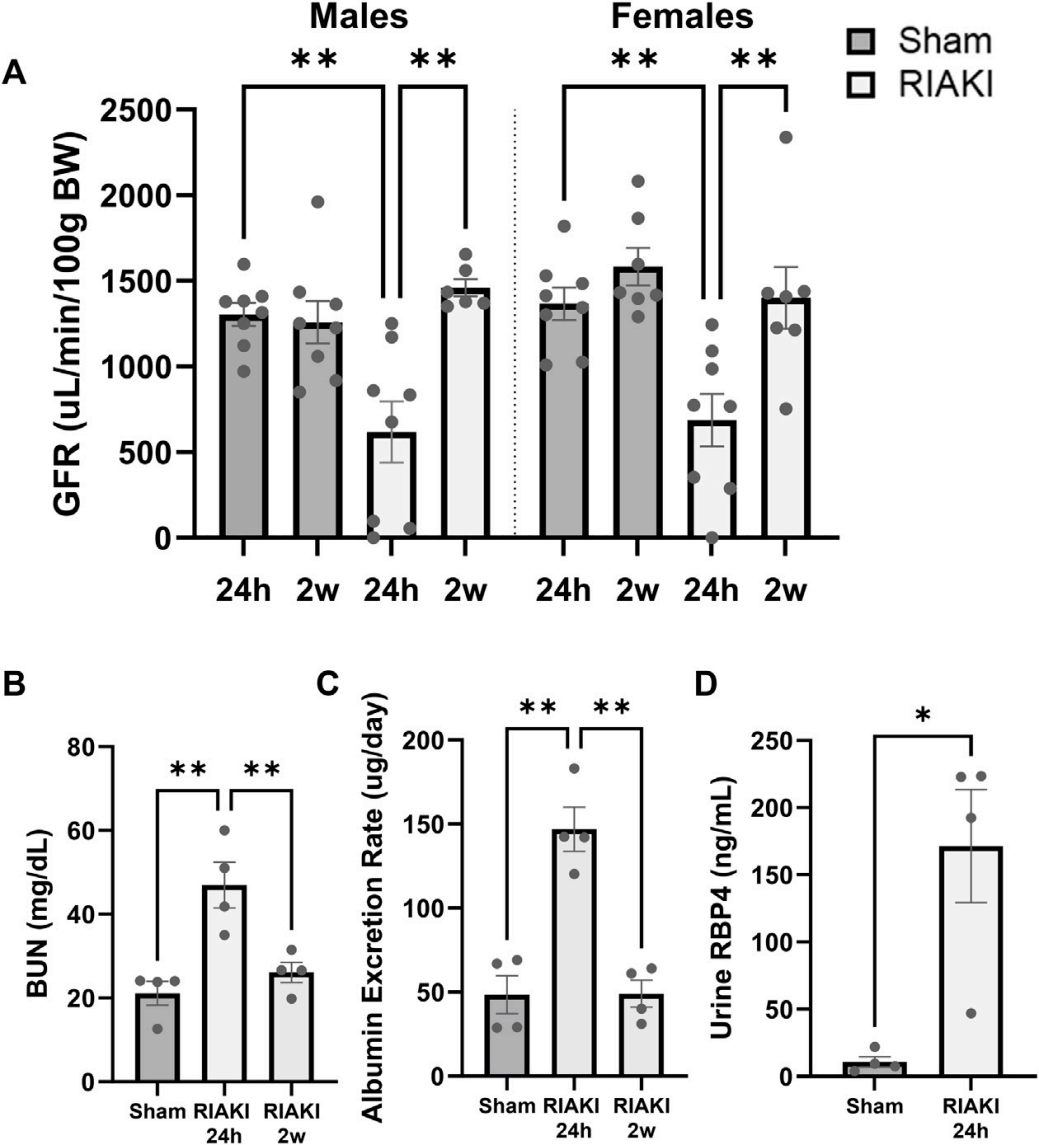
The glycerol injection model of rhabdomyolysis-induced acute kidney injury (RIAKI) caused transient renal functional impairment, which was reversed by 2 weeks. **(A)** Glomerular filtration rate (GFR; uL/min/100 g body weight [BW]) of the control versus RIAKI male (left) and female (right) mice. The GFR is significantly reduced after rhabdomyolysis but recovers 2 weeks post-injury. **(B)** High levels of blood urea nitrogen (BUN) and **(C)** urinary albumin were observed following RIAKI. BUN and urinary albumin levels returned to baseline by 2 weeks. **(D)** The urinary retinol-binding protein 4 (RBP4) level, a marker of proximal tubule dysfunction, was increased significantly 24 h following RIAKI. One-way ANOVA with *post hoc* tests for multiple-group analyses and the t-test for two-group analyses were conducted. *: *p* < 0.05. **: *p* < 0.01.

First, we characterized RIAKI and its resolution in male and female mice. Here, 24 h after the induction of rhabdomyolysis, the male and female mice had a reduced GFR compared to the sex-matched controls; the GFR returned to baseline by 2 weeks post-rhabdomyolysis (Figure 1A). The blood urea nitrogen level was increased 24 h following rhabdomyolysis but returned to baseline within 2 weeks (Figure 1B), with similar results observed in urine albumin concentrations (Figure 1C). RBP4 is a small protein associated with proximal tubule dysfunction when found in the urine (Norden et al., 2014); it significantly increased 24 h following rhabdomyolysis (Figure 1D). These data confirm the development of AKI after glycerol injection and recovery from AKI before breeding.

### RIAKI affects fetal and perinatal outcomes but not fertility

**FIGURE 2:**
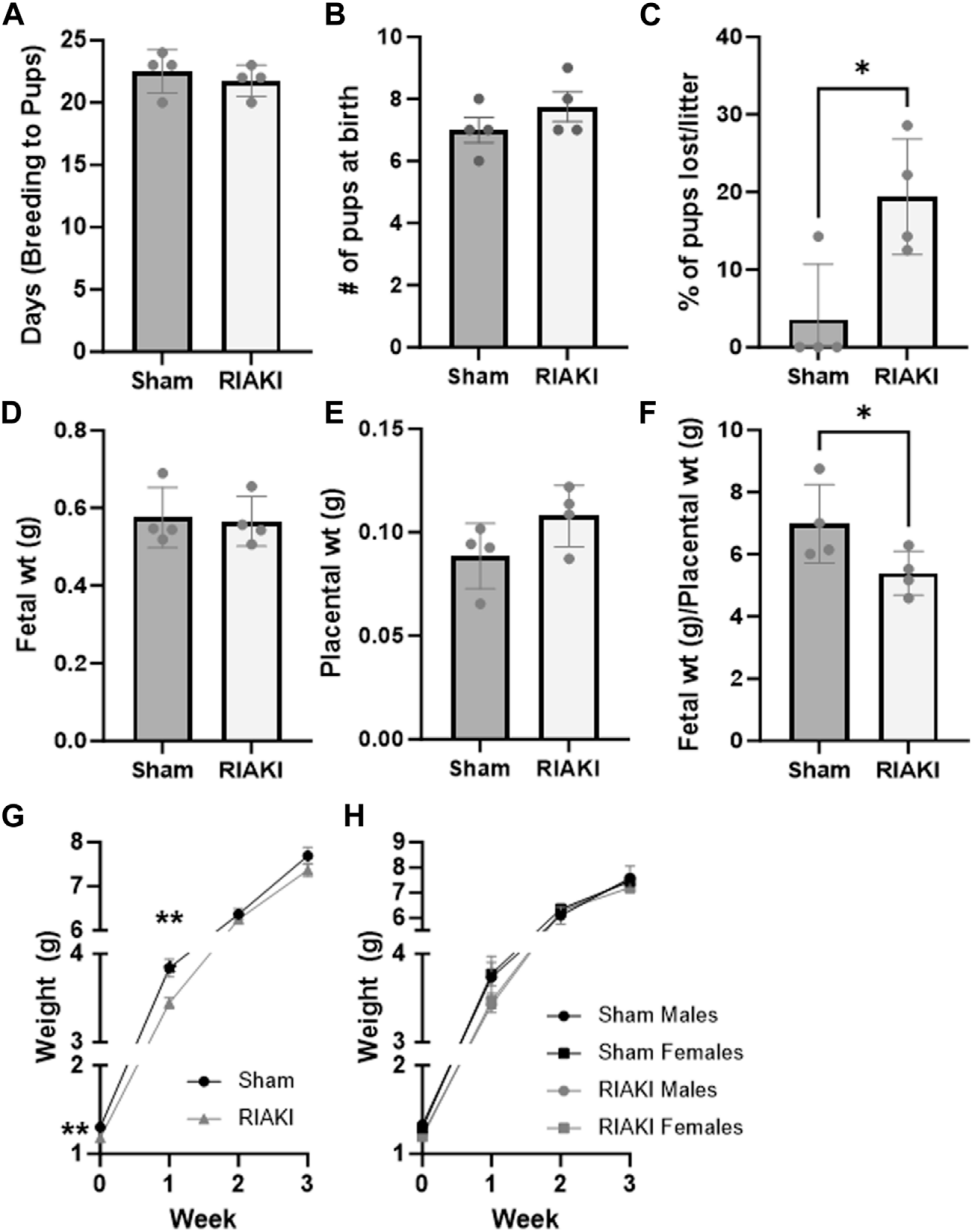
Recovered parental RIAKI causes fetal growth restriction but does not affect fertility. RIAKI did not affect fertility, as measured by **(A)** the number of days from pair introduction to birth and **(B)** litter sites. **(C)** More pups from RIAKI pairings died in the perinatal period than pups from sham pairings. Neither **(D)** fetal nor **(E)** placental weight was significantly different, but **(F)** placental sufficiency (fetal weight/placental weight) decreased in pups from RIAKI pairings. **(G)** Pups from RIAKI pairs were born smaller and remained smaller in the first week than pups from shams. By 2 weeks, the weights of the pups were not different between groups. **(H)** Fetal sex was not a factor in differences. Litters: *n* = 4. **(D–F)** Presented as the average per litter. **(A–G)** T-test for two-group analyses. **(H)** One-way ANOVA with *post hoc* tests. *: *p* < 0.05. **: *p* < 0.01.

Next, we evaluated the effect of RIAKI on fertility and perinatal outcomes. Sham and RIAKI matings demonstrated similar time to conception after pairing (Figure 2A). No difference in weights between dams on GD 16.5 (term 19.5) was observed (sham: 34.03 g ± 2.69 g; RIAKI: 34.62 ± 3.00 g; NS). Additionally, initial litter sizes at birth were the same in both groups (Figure 2B). However, pups from RIAKI pairings had a 3.14 ± 0.50 times higher relative risk of death in the perinatal (birth to weaning) period than pups from sham pregnancies, with approximately ¼ of pups from RIAKI pairing litters dying before weaning (Figure 2C). Although fetal weights (Figure 2D) and placental weights (Figure 2E) were not significantly different between the groups, the ratio of the fetal weight to placental weight was 16.8% lower in pups from RIAKI pairs, indicating placental insufficiency and fetal growth restriction (Figure 2F). Birthweights were 5.0% lower in RIAKI pups, and pups grew less quickly in the first week, although weights caught up by 2 weeks of age and remained similar through 12 weeks (Figures 2G, 4). This difference in fetal weight was not mediated by sex; males and females from RIAKI pairings were significantly smaller at 1 week (Figure 2H). We conclude that RIAKI pairings did not experience reduced fertility but resulted in growth-restricted pups with higher perinatal mortality than those from sham pairings.

### Recovered parental RIAKI causes abnormal pregnancy

**FIGURE 3:**
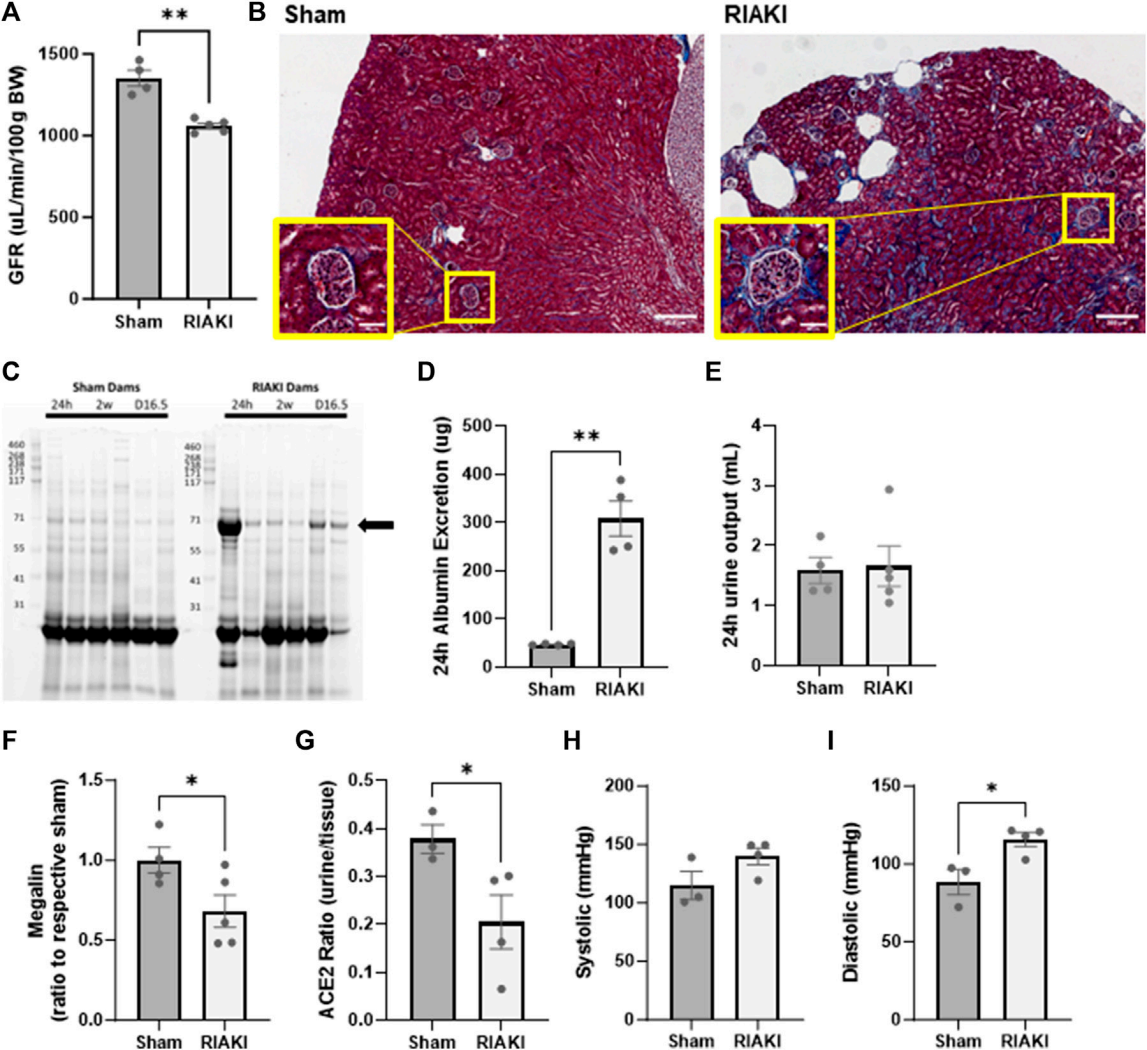
Exposure to recovered RIAKI results in intrapregnancy renal functional and histological derangement at gestational day (GD) 16.5. **(A)** The GFR was selectively induced in recovered RIAKI-exposed dams, accompanied by **(B)** new subcapsular fibrosis (see insets). **(C)** RIAKI dams also exhibited low-molecular weight proteinuria in urine gels. The marked band was suspected to be albumin; we confirmed that the total urinary excretion of albumin over 24 h was elevated **(D)**. Urine excretion amounts were not different between groups over 24 h **(E)**. Assessment of **(F)** tubular megalin confirms that recovered RIAKI was correlated with the reduction in tubular megalin levels. **(G)** The ratio of urine to tissue ACE2 activity is reduced, confirming a change in renal renin–angiotensin system (RAS) components. No change was observed in **(H)** systolic blood pressure during late-term pregnancy, but **(I)** diastolic blood pressure was higher in recovered RIAKI dams. T-test for two groups. *: *p* < 0.05. **: *p* < 0.01.

To evaluate the influence of recovered RIAKI on maternal and intrauterine physiology, we evaluated renal function, blood pressure, and select components of the intra-renal RAS at GD 16.5. This time point is considered late in pregnancy when the fetus is still in a period of rapid growth but after the placenta has reached its maximum development (Coan et al., 2004). Although renal functional recovery was evident in parents 2 weeks following RIAKI, the GFR in RIAKI-bred dams was reduced compared to sham dams (Figure 3A). Masson’s trichrome-stained histological sections showed evidence of subcapsular fibrosis in RIAKI dams that was absent in sham dams (Figure 3B). Furthermore, 24-h urine protein excretion was increased in RIAKI dams 24 h following rhabdomyolysis, which was resolved in 2 weeks, but proteinuria returned in these dams by GD 16.5, particularly low-molecular weight proteinuria (Figure 3C). Measuring the urine albumin content by ELISA confirmed albuminuria in pregnancy following recovered AKI seven times greater than that of sham dams (Figure 3D); this was driven by the albumin concentration and not urine volume (Figure 3E). A Western blot for megalin on proteins isolated from the renal brush border revealed lower megalin expression in RIAKI dams than in shams (Figure 3F; Supplementary Figure S1), supporting a conclusion of unrecovered or pregnancy-exacerbated proximal tubule damage following RIAKI. Assessment of renin–angiotensin system factors in RIAKI dams revealed a reduced angiotensin-converting enzyme 2 (ACE2) urine:tissue activity ratio (Figure 3G), suggesting RAS imbalance in the kidney. Dams showed no change in systolic pressure, but diastolic blood pressure was elevated in recovered RIAKI dams (Figures 3H, I). Together, these data indicate that pregnancies in recovered RIAKI dams are complicated by renal dysfunction caused by fibrosis and loss of tubular function, accompanied by alterations in blood pressure.

### Offspring of recovered RIAKI parents develop renal dysfunction and gain weight in adulthood

**FIGURE 4:**
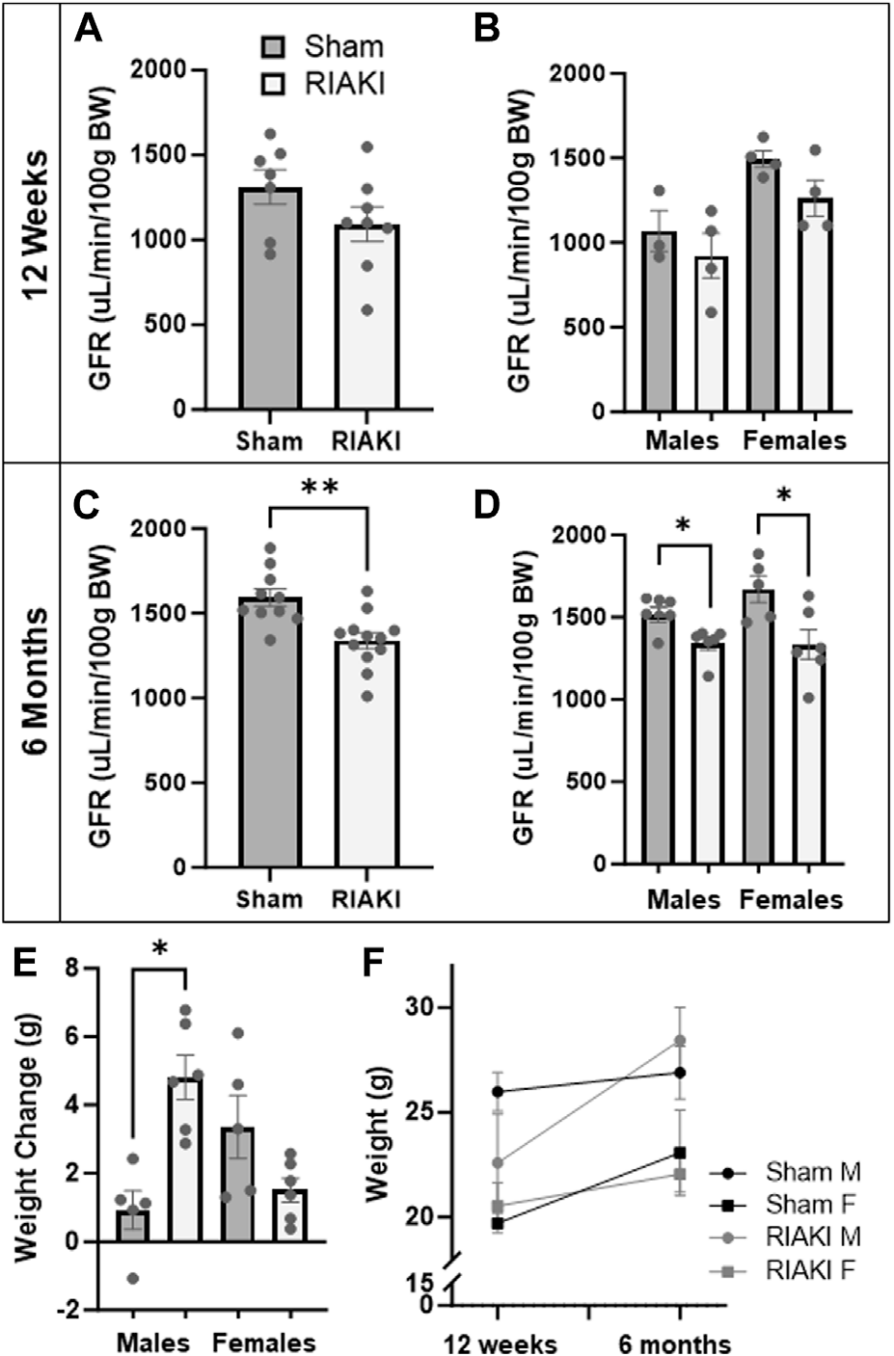
Adult offspring of recovered RIAKI parents develop renal dysfunction with age. **(A)** The GFR of 12-week-old offspring was not significantly different, nor was **(B)** sex a factor. **(C)** By 6 months old, the GFR was reduced in all RIAKI offspring, **(D)** regardless of sex. **(E)** Between young (12 weeks) and middle (6 months) adulthood, males from RIAKI pairings gained significantly more weight than sham males, **(F)** with significantly steeper weight gain slopes (male slopes *p* = 0.005). T-test for two groups and one-way ANOVA with *post hoc* tests for multiple comparisons. *: *p* < 0.05. **: *p* < 0.01.

Next, to determine the inter-generational impact of parental AKI, we performed a longitudinal evaluation of the surviving offspring. Young adult offspring (12 weeks) of RIAKI dams did not have a significantly lower GFR than offspring of shams (Figure 4A; *p* = 0.12), a trend that did not change when data were separated by sex (Figure 4B). However, by middle age (6 months), the GFR was 17.4% lower in RIAKI adult offspring by group (Figure 4C) in both sexes (Figure 4D). Males from RIAKI parents gained more weight between young adulthood and middle age than shams, while females were not significantly different based on parentage (Figure 4E); this is reflected by a steeper weight gain slope, with males differing significantly (*p* = 0.005, Figure 4F). These data suggest that offspring may be developmentally programmed for adult-onset renal dysfunction by parental recovered RIAKI despite not being exposed to RIAKI themselves; moreover, male offspring of RIAKI pairings may be metabolically challenged in later life.

### Perturbations of the RAS in early adulthood are not reflected in older adult blood pressures

**FIGURE 5:**
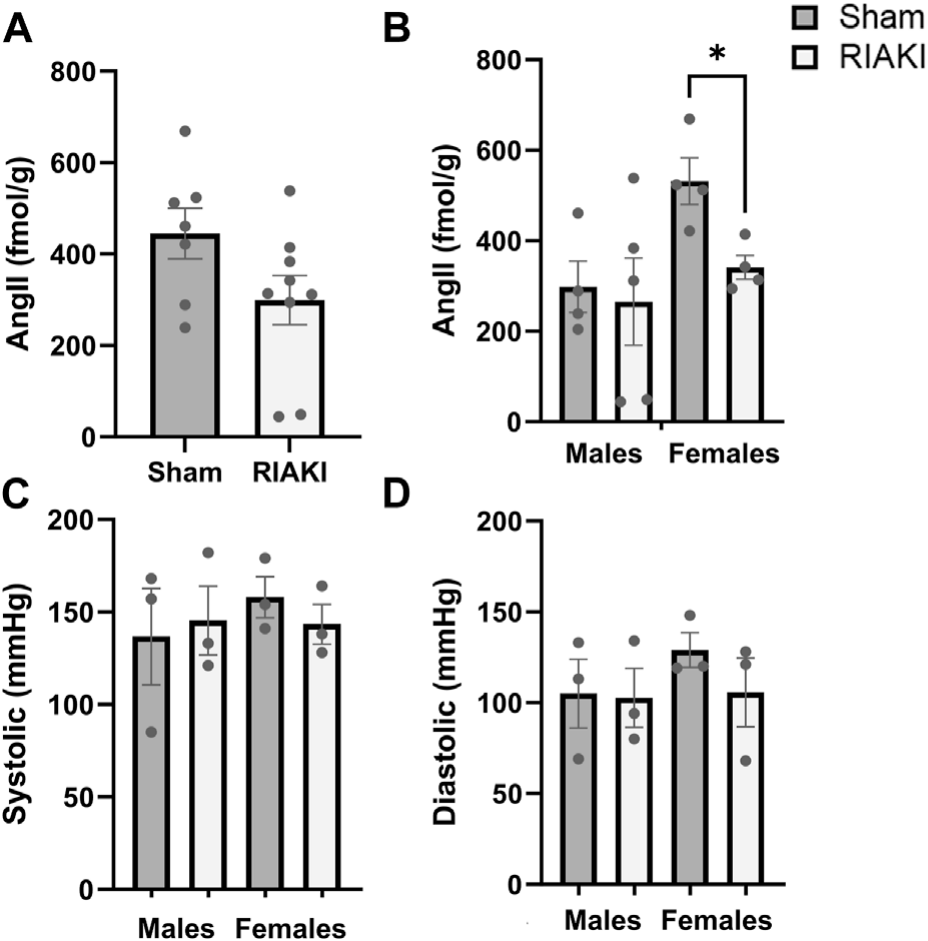
RAS but not blood pressure is affected in adult offspring of sham and RIAKI parents. **(A)** In 12-week-old offspring, AngII kidney tissue content varied by breeding pairing and **(B)** offspring sex. Females from sham pairings had higher AngII levels than males; female offspring of RIAKI had lower AngII levels than sham females. However, **(C)** systolic and **(D)** diastolic blood pressures of 6-month-old offspring were not significantly different. T-test for two groups and one-way ANOVA with *post hoc* tests. *: *p* < 0.05.

Lastly, we evaluated the components of the intra-renal RAS in offspring. Measurements of renal AngII levels in sham and RIAKI offspring at 12 weeks old were not significantly different based on parentage alone (Figure 5A), but females from RIAKI dams had 43.6% lower AngII levels than female offspring of sham dams (Figure 5B). No apparent impact on blood pressure metrics was observed at 6 months: systolic (Figure 5C) and diastolic blood pressures (Figure 5D) were unchanged when analyzed with or without regard to separation by sex. This suggests that while the vasoconstrictive AngII level is reduced in female pups from RIAKI pairings in early adulthood, no changes in adult blood pressure are evident in RIAKI offspring.

## Discussion

In an AKI model relevant to reproductive health, we show that parents who have recovered from AKI nonetheless cause immediate and lifelong risks to their offspring. Despite recovery from AKI, we found that these dams develop new kidney disease during pregnancy, accompanied by increased blood pressure and an altered intra-renal RAS. Adult offspring of these dams developed delayed-onset renal and metabolic abnormalities. These findings likely resulted from parental systemic or renal disease, not reproductive system failure, since parental RIAKI did not alter fertility: RIAKI- and sham-exposed parents produced the same number of pups at the same interval. However, the offspring of RIAKI parents were small for their gestational age, more likely to die, and gained weight less rapidly than those from sham parents. This was accompanied by placental insufficiency, as measured by the fetal-to-placental weight ratio near term. Clinical studies have demonstrated an association between prior AKI and preeclampsia (Tangren et al., 2017; Tangren et al., 2018). Furthermore, using the renal ischemia–reperfusion injury AKI model, Gillis et al. (2021) first characterized recovered AKI-induced fetal growth restriction with normal fertility and described impaired placental blood flow and altered antepartum maternal hemodynamics and renal sodium handling. Our findings corroborate those of Gillis et al.; we use a similar model of AKI that occurs in humans of reproductive age. Additionally, our study adds a novel understanding of the connection of the antepartum maternal kidney to the uterine environment to connect maternal and offspring disease pathogenesis.

In our AKI model, we investigated the maternal renal function and the intra-renal RAS, which plays a role in sodium and hemodynamic regulation (Pohl et al., 2010; Bullen et al., 2023). The evaluation of antepartum maternal renal function, a principal determinant of the intrauterine environment, revealed surprising late-pregnancy GFR loss accompanied by tubular proteinuria and renal fibrosis. The observation of tubular proteinuria was striking and may be an important mechanistic insight. Pre-pregnancy RIAKI was accompanied by urine RBP4 elevation. RBP4 is a megalin ligand (Norden et al., 2014) and, in the presence of tubular proteinuria, confirms the impairment of megalin-dependent function in the proximal tubule (Bullen et al., 2023). The recurrence of GFR loss in pregnancy was coupled with reduced megalin expression, perhaps suggesting incomplete or maladaptive repair in the proximal tubule following acute kidney injury despite the outward appearance of recovery. As angiotensinogen and AngII bind and are internalized by megalin, and brush border ACE2 distribution is regulated by megalin (Pohl et al., 2010), we evaluated renal ACE2 activity and AngII content. Our AngII findings were inconclusive (Supplementary Figure S2), but antepartum megalin loss was accompanied by a reduced ratio of urine–brush border ACE2 activity, which we speculate may be a compensatory effect to attempt to conserve proximal tubule ACE2 levels. These data suggest that lasting or recurring disruption of megalin function by RIAKI dysregulates the intra-renal RAS in pregnancy.

Normal pregnancy relies on a balance of factors to preserve cardiovascular function, many of which are controlled by the kidney [reviewed by Touyz and Montezano (2018)]. We observed diastolic blood pressure elevation in recovered RIAKI dams. We speculate that elevated blood pressure may result from RAS imbalance or increased renal (or plasma) AngII levels affecting the vascular tone. Further mechanistic study should focus on untangling the interactions between renal megalin and ACE2 during pregnancy and the resultant effects on systemic AngII blood pressure, sodium homeostasis, and fetal development.

Some of our findings may be seen as echoing aspects of human preeclampsia, in which albuminuria, hypertension, and RAS abnormalities are associated with fetal growth restriction and poor fetal outcomes. In distinction from preeclampsia, however, we did not find significantly elevated maternal systolic blood pressure. Tubular proteinuria, as opposed to glomerular proteinuria, and the absence of glomerular lesions on kidney sections argue against the development of endotheliopathy, which is a principal underpinning of preeclampsia. Therefore, the syndrome observed in our mouse model more closely resembles observations in mild or subclinical renal impairment: combined systolic and diastolic hypertension and endotheliopathy are not present during such pregnancies, but the risk of preterm birth and fetal growth restriction is increased (Hladunewich et al., 2016). Since tight control of diastolic pressure during pregnancy in patients with hypertension is effective at controlling overall maternal hypertension (Magee et al., 2015), and the treatment of maternal hypertension may improve fetal and maternal outcomes (Dimitriadis et al., 2023), our preclinical study and related work may ultimately have translational implications for prenatal screening and care.

Since adverse pregnancies can program offspring for adult-onset disease (Barker et al., 1989; Barker, 1990; Newnham, 2007; Rodrguez-Rodrguez et al., 2018; Lu and Hu, 2019), we considered long-term developmental programming consequences of parental RIAKI. We followed the offspring of RIAKI and sham pairings through adulthood. At 6 months old, we observed no change in blood pressure in the offspring of RIAKI-exposed parents, but they demonstrated a reduced GFR regardless of sex and altered weight gain exclusively in males. Although young adult offspring showed normal renal function, the renal angiotensin II level was reduced in the progeny of RIAKI parents, which may be an early warning of future renal dysfunction. This was entirely driven by the female sex: the female offspring of RIAKI parents halved their AngII level. Other studies of RAS balance have found that estrogen stimulates ACE2 expression and that males with CKD have lower ACE2 glomerular expression than females (Shoemaker et al., 2019; Maksimowski et al., 2020). The observed reduction in AngII levels in young adult females might represent a compensatory response to preventing higher blood pressure, plasma sodium levels, or GFR. Alterations in the RAS in female offspring may help avoid some of the fate that befalls their male siblings.

Our study has limitations. First, our mouse model findings may not fully extend to human diseases. Because both paternal and maternal influence may determine fetal development, we studied parings in which both parents were exposed to sham or RIAKI. Subsequently, we cannot exclude paternal involvement in the observed effects or the necessity of exposing both parents to RIAKI. Further studies are necessary to determine whether maternal RIAKI is sufficient. We are currently investigating an isolated maternal post-RIAKI model with no paternal or fetal RIAKI influence to expand our studies. Similarly, we cannot exclude rearing behavior or efficient nutrient availability during the peripartum period as a cause of adult disease; further study will be required to elucidate the role of this vital component in development. The weight change observed in the adult male offspring of RIAKI parents is a valuable observation; although we do not currently know whether this is due to edema or obesity, both would point toward dysfunctional programming during development. Metabolic studies of offspring are planned to differentiate the causes of weight gain. Finally, blood pressures were measured in awake mice using tail plethysmography, which may have lacked sensitivity to detect subtle changes in blood pressure resulting from prior parental AKI. Although our studies were powered to examine the differences based on the GFR, the results generated by our blood pressure studies are hypothesis-generating and promising pilot data for future studies.

We conclude that parental recovered RIAKI causes impaired fetal development and maternal antepartum kidney disease reflected in megalin loss and an impaired intra-renal RAS. These data demonstrate both similarities and differences with characteristics of preeclampsia, a major clinical problem. Our data also implicate parental RIAKI in adult kidney disease of the offspring, suggesting a novel potential mechanism underpinning the developmental origins of the disease.

## Data Availability

The raw data supporting the conclusion of this article will be made available by the authors, without undue reservation.

## References

[B1] Abdel-Kader K. Palevsky P. M. (2009). Acute kidney injury in the elderly. Clin. Geriatr. Med. 25 (3), 331–358. 10.1016/j.cger.2009.04.001 19765485 PMC2748997

[B2] Barker D. J. (1990). The fetal and infant origins of adult disease. BMJ Clin. Res. 301 (6761), 1111. 10.1136/bmj.301.6761.1111 PMC1664286 2252919

[B3] Barker D. J. Osmond C. Golding J. Kuh D. Wadsworth M. E. (1989). Growth *in utero*, blood pressure in childhood and adult life, and mortality from cardiovascular disease. BMJ Clin. Res. 298 (6673), 564–567. 10.1136/bmj.298.6673.564 PMC1835925 2495113

[B4] Biber J. Stieger B. Haase W. Murer H. (1981). A high yield preparation for rat kidney brush border membranes Different behaviour of lysosomal markers. Biochimica Biophysica Acta (BBA) - Biomembr. 647 (2), 169–176. 10.1016/0005-2736(81)90243-1 6117319

[B5] Bullen A. L. Fregoso A. Ascher S. B. Shlipak M. G. Ix J. H. Rifkin D. E. (2023). Markers of kidney tubule dysfunction and major adverse kidney events. Nephron. 147, 713–716. 10.1159/000531946 37524063

[B6] Chawla L. S. Amdur R. L. Shaw A. D. Faselis C. Palant C. E. Kimmel P. L. (2014). Association between AKI and long-term renal and cardiovascular outcomes in United States veterans. Clin. J. Am. Soc. Nephrol. 9 (3), 448–456. 10.2215/CJN.02440213 24311708 PMC3944753

[B7] Coan P. M. Ferguson-Smith A. C. Burton G. J. (2004). Developmental dynamics of the definitive mouse placenta assessed by stereology. Biol. reproduction 70 (6), 1806–1813. 10.1095/biolreprod.103.024166 14973263

[B8] Deng J. Hu X. Yuen P. S. Star R. A. (2004). Alpha-melanocyte-stimulating hormone inhibits lung injury after renal ischemia/reperfusion. Am. J. Respir. Crit. Care Med. 169 (6), 749–756. 10.1164/rccm.200303-372OC 14711793

[B9] Dimitriadis E. Rolnik D. L. Zhou W. Estrada-Gutierrez G. Koga K. Francisco R. P. V. (2023). Pre-eclampsia. Nat. Rev. Dis. Prim. 9 (1), 8. 10.1038/s41572-023-00417-6 36797292

[B10] Dumanski S. M. Ahmed S. B. (2019). Fertility and reproductive care in chronic kidney disease. J. Nephrol. 32 (1), 39–50. 10.1007/s40620-018-00569-9 30604149

[B11] Eberle C. Kirchner M. F. Herden R. Stichling S. (2020). Paternal metabolic and cardiovascular programming of their offspring: a systematic scoping review. PLoS One 15 (12), e0244826. 10.1371/journal.pone.0244826 33382823 PMC7775047

[B12] Elterman J. Zonies D. Stewart I. Fang R. Schreiber M. (2015). Rhabdomyolysis and acute kidney injury in the injured war fighter. J. Trauma Acute Care Surg. 79 (4), S171–S174. 10.1097/TA.0000000000000572 26131786

[B13] Galaviz-Hernandez C. Sosa-Macias M. Teran E. Garcia-Ortiz J. E. Lazalde-Ramos B. P. (2019). Paternal determinants in preeclampsia. Front. Physiology 9 (1870), 1870. 10.3389/fphys.2018.01870 PMC6330890 30666213

[B14] Gameiro J. Fonseca J. A. Outerelo C. Lopes J. A. (2020). Acute kidney injury: from diagnosis to prevention and treatment strategies. J. Clin. Med. 9 (6), 1704. 10.3390/jcm9061704 32498340 PMC7357116

[B15] Garovic V. D. Dechend R. Easterling T. Karumanchi S. A. McMurtry Baird S. Magee L. A. (2022). Hypertension in pregnancy: diagnosis, blood pressure goals, and pharmacotherapy: a scientific statement from the American heart association. Hypertension 79 (2), e21–e41. 10.1161/HYP.0000000000000208 34905954 PMC9031058

[B16] Gburek J. Birn H. Verroust P. J. Goj B. Jacobsen C. Moestrup S. K. (2003). Renal uptake of myoglobin is mediated by the endocytic receptors megalin and cubilin. Am. J. Physiology-Renal Physiology 285 (3), F451–F458. 10.1152/ajprenal.00062.2003 12724130

[B17] Gillis E. E. Brands M. W. Sullivan J. C. (2021). Adverse maternal and fetal outcomes in a novel experimental model of pregnancy after recovery from renal ischemia-reperfusion injury. J. Am. Soc. Nephrol. 32 (2), 375–384. 10.1681/ASN.2020020127 33408137 PMC8054890

[B18] Harville E. W. Catov J. Lewis C. E. Bibbins-Domingo K. Gunderson E. P. (2019). Pre-pregnancy kidney function and subsequent adverse pregnancy outcomes. Pregnancy Hypertens. 15, 195–200. 10.1016/j.preghy.2019.01.011 30825922 PMC6484837

[B19] Hebert J. F. Funahashi Y. Hutchens M. P. (2023). Harm! foul! How acute kidney injury SHReDDs patient futures. Curr. Opin. Nephrol. Hypertens. 32 (2), 165–171. 10.1097/MNH.0000000000000864 36683541 PMC10079264

[B20] Hebert J. F. Millar J. A. Raghavan R. Romney A. Podrabsky J. E. Rennie M. Y. (2021). Male fetal sex affects uteroplacental angiogenesis in growth restriction mouse model. Biol. Reproduction 104 (4), 924–934. 10.1093/biolre/ioab006 PMC8023425 33459759

[B21] Hladunewich M. A. Melamed N. Bramham K. (2016). Pregnancy across the spectrum of chronic kidney disease. Kidney Int. 89 (5), 995–1007. 10.1016/j.kint.2015.12.050 27083278

[B22] Holley J. L. Schmidt R. J. (2013). Changes in fertility and hormone replacement therapy in kidney disease. Adv. Chronic Kidney Dis. 20 (3), 240–245. 10.1053/j.ackd.2013.01.003 23928388

[B23] Hummel K. Gregory A. Desai N. Diamond A. (2016). Rhabdomyolysis in adolescent athletes: review of cases. Phys. Sportsmed. 44 (2), 195–199. 10.1080/00913847.2016.1170582 27031535

[B24] James M. T. Ghali W. A. Knudtson M. L. Ravani P. Tonelli M. Faris P. (2011). Associations between acute kidney injury and cardiovascular and renal outcomes after coronary angiography. Circulation 123 (4), 409–416. 10.1161/CIRCULATIONAHA.110.970160 21242477

[B25] Kelly K. J. (2003). Distant effects of experimental renal ischemia/reperfusion injury. J. Am. Soc. Nephrol. 14 (6), 1549–1558. 10.1097/01.asn.0000064946.94590.46 12761255

[B26] Kim D. J. Park S. H. Sheen M. R. Jeon U. S. Kim S. W. Koh E. S. (2006). Comparison of experimental lung injury from acute renal failure with injury due to sepsis. Respiration 73 (6), 815–824. 10.1159/000095588 16960438

[B27] Kodadek L. Carmichael S. P. Seshadri A. Pathak A. Hoth J. Appelbaum R. (2022). Rhabdomyolysis: an American association for the surgery of trauma critical care committee clinical consensus document. Trauma Surg. Acute Care Open 7 (1), e000836. 10.1136/tsaco-2021-000836 35136842 PMC8804685

[B28] Lameire N. Van Biesen W. Hoste E. Vanholder R. (2008). The prevention of acute kidney injury: an in-depth narrative review Part 1: volume resuscitation and avoidance of drug- and nephrotoxin-induced AKI. NDT Plus 1, 392–402. 10.1093/ndtplus/sfn162 28657002 PMC5477885

[B29] Liu M. Liang Y. Chigurupati S. Lathia J. D. Pletnikov M. Sun Z. (2008). Acute kidney injury leads to inflammation and functional changes in the brain. J. Am. Soc. Nephrol. 19 (7), 1360–1370. 10.1681/ASN.2007080901 18385426 PMC2440297

[B30] Loutradis C. Pickup L. Law J. P. Dasgupta I. Townend J. N. Cockwell P. (2021). Acute kidney injury is more common in men than women after accounting for socioeconomic status, ethnicity, alcohol intake and smoking history. Biol. Sex Differ. 12 (1), 30. 10.1186/s13293-021-00373-4 33832522 PMC8034098

[B31] Lu H. Q. Hu R. (2019). Lasting effects of intrauterine exposure to preeclampsia on offspring and the underlying mechanism. AJP Rep. 9 (3), e275–e291. 10.1055/s-0039-1695004 31511798 PMC6736667

[B32] Lumbers E. R. Delforce S. J. Arthurs A. L. Pringle K. G. (2019). Causes and consequences of the dysregulated maternal renin-angiotensin system in preeclampsia. Front. Endocrinol. 10, 563. 10.3389/fendo.2019.00563 PMC6746881 31551925

[B33] Lundy S. D. Vij S. C. (2019). Male infertility in renal failure and transplantation. Transl. Androl. Urol. 8 (2), 173–181. 10.21037/tau.2018.07.16 31080778 PMC6503227

[B34] Magee L. A. von Dadelszen P. Rey E. Ross S. Asztalos E. Murphy K. E. (2015). Less-tight versus tight control of hypertension in pregnancy. N. Engl. J. Med. 372 (5), 407–417. 10.1056/NEJMoa1404595 25629739

[B35] Makris K. Spanou L. (2016). Acute kidney injury: definition, Pathophysiology and clinical phenotypes. Clin. Biochem. Rev. 37 (2), 85–98. 28303073 PMC5198510

[B36] Maksimowski N. Williams V. R. Scholey J. W. (2020). Kidney ACE2 expression: implications for chronic kidney disease. PLoS One 15 (10), e0241534. 10.1371/journal.pone.0241534 33125431 PMC7598523

[B37] Matsushita K. Mori K. Saritas T. Eiwaz M. Funahashi Y. Nickerson M. (2021). Cilastatin ameliorates rhabdomyolysis-induced AKI in mice. J. Am. Soc. Nephrol. 32 (10), 2579–2594. 10.1681/ASN.2020030263 34341182 PMC8722809

[B38] Miyazawa S. Watanabe H. Miyaji C. Hotta O. Abo T. (2002). Leukocyte accumulation and changes in extra-renal organs during renal ischemia reperfusion in mice. J. Lab. Clin. Med. 139 (5), 269–278. 10.1067/mlc.2002.122832 12032487

[B39] Nadim M. K. Forni L. G. Mehta R. L. Connor M. J. Liu K. D. Ostermann M. (2020). COVID-19-associated acute kidney injury: consensus report of the 25th Acute Disease Quality Initiative (ADQI) Workgroup. Nat. Rev. Nephrol. 16 (12), 747–764. 10.1038/s41581-020-00356-5 33060844 PMC7561246

[B40] Newnham J. P. (2007). The developmental origins of health and disease (DOHaD) – why it is so important to those who work in fetal medicine. Ultrasound Obstet. Gynecol. 29, 121–123. 10.1002/uog.3938 17252533

[B41] Norden A. G. Lapsley M. Unwin R. J. (2014). Urine retinol-binding protein 4: a functional biomarker of the proximal renal tubule. Adv. Clin. Chem. 63, 85–122. 10.1016/b978-0-12-800094-6.00003-0 24783352

[B42] Palant C. E. Amdur R. L. Chawla L. S. (2017). Long-term consequences of acute kidney injury in the perioperative setting. Curr. Opin. Anesthesiol. 30 (1), 100–104. 10.1097/ACO.0000000000000428 27977430

[B43] Perico N. Askenazi D. Cortinovis M. Remuzzi G. (2018). Maternal and environmental risk factors for neonatal AKI and its long-term consequences. Nat. Rev. Nephrol. 14 (11), 688–703. 10.1038/s41581-018-0054-y 30224767

[B44] Petejova N. Martinek A. (2014). Acute kidney injury due to rhabdomyolysis and renal replacement therapy: a critical review. Crit. Care 18 (3), 224. 10.1186/cc13897 25043142 PMC4056317

[B45] Piccoli G. B. Alrukhaimi M. Liu Z.-H. Zakharova E. Levin A. World Kidney Day Steering Committee (2018). Women and kidney diseases: questions unanswered and answers unquestioned. Kidney Int. Rep. 3 (2), 225–235. 10.1016/j.ekir.2018.01.001 29725625 PMC5932302

[B46] Pohl M. Kaminski H. Castrop H. Bader M. Himmerkus N. Bleich M. (2010). Intrarenal renin angiotensin system revisited: role of megalin-dependent endocytosis along the proximal nephron. J. Biol. Chem. 285 (53), 41935–41946. 10.1074/jbc.M110.150284 20966072 PMC3009920

[B47] Rodrguez-Rodrguez P. Ramiro-Cortijo D. Reyes-Hernndez C. G. Lopez de Pablo A. L. Gonzalez M. C. Arribas S. M. (2018). Implication of oxidative stress in fetal programming of cardiovascular disease. Front. Physiol. 9, 602. 10.3389/fphys.2018.00602 29875698 PMC5974054

[B48] Scarfe L. Schock-Kusch D. Ressel L. Friedemann J. Shulhevich Y. Murray P. (2018). Transdermal measurement of glomerular filtration rate in mice. J. Vis. Exp. 140, 58520. 10.3791/58520 PMC6235579 30394397

[B49] Schiffl H. (2020). Gender differences in the susceptibility of hospital-acquired acute kidney injury: more questions than answers. Int. Urology Nephrol. 52 (10), 1911–1914. 10.1007/s11255-020-02526-7 PMC7515943 32661623

[B50] Shoemaker R. Tannock L. R. Su W. Gong M. Gurley S. B. Thatcher S. E. (2019). Adipocyte deficiency of ACE2 increases systolic blood pressures of obese female C57BL/6 mice. Biol. Sex. Differ. 10 (1), 45. 10.1186/s13293-019-0260-8 31484552 PMC6727421

[B51] Silver S. A. Chertow G. M. (2017). The economic consequences of acute kidney injury. Nephron 137 (4), 297–301. 10.1159/000475607 PMC5743773 28595193

[B52] Stewart I. J. Faulk T. I. Sosnov J. A. Clemens M. S. Elterman J. Ross J. D. (2016). Rhabdomyolysis among critically ill combat casualties: associations with acute kidney injury and mortality. J. Trauma Acute Care Surg. 80 (3), 492–498. 10.1097/TA.0000000000000933 26670111

[B53] Suarez M. L. G. Kattah A. Grande J. P. Garovic V. (2019). Renal disorders in pregnancy: Core curriculum 2019. Am. J. Kidney Dis. 73 (1), 119–130. 10.1053/j.ajkd.2018.06.006 30122546 PMC6309641

[B54] Tangren J. S. Powe C. E. Ankers E. Ecker J. Bramham K. Hladunewich M. A. (2017). Pregnancy outcomes after clinical recovery from AKI. J. Am. Soc. Nephrol. 28 (5), 1566–1574. 10.1681/ASN.2016070806 28008002 PMC5407730

[B55] Tangren J. S. Wan M. Adnan W. A. H. Powe C. E. Ecker J. Bramham K. (2018). Risk of preeclampsia and pregnancy complications in women with a history of acute kidney injury. Hypertension 72 (2), 451–459. 10.1161/HYPERTENSIONAHA.118.11161 29915020 PMC6074052

[B56] Touyz R. M. Montezano A. C. (2018). Angiotensin-(1-7) and vascular function: the clinical context. Hypertension 71 (1), 68–69. 10.1161/HYPERTENSIONAHA.117.10406 29203630

[B57] Turgut F. Awad A. S. Abdel-Rahman E. M. (2023). Acute kidney injury: medical causes and pathogenesis. J. Clin. Med. 12 (1), 375. 10.3390/jcm12010375 36615175 PMC9821234

[B58] Wang X. Ji X. (2020). Sample size estimation in clinical research: from randomized controlled trials to observational studies. Chest 158 (1), S12–S20. 10.1016/j.chest.2020.03.010 32658647

[B59] Wei Q. Hill W. D. Su Y. Huang S. Dong Z. (2011). Heme oxygenase-1 induction contributes to renoprotection by G-CSF during rhabdomyolysis-associated acute kidney injury. Am. J. Physiology - Ren. Physiology 301 (1), F162–F170. 10.1152/ajprenal.00438.2010 PMC3129892 21511696

[B60] Whitten W. K. (1956). Modification of the oestrous cycle of the mouse by external stimuli associated with the male. J. Endocrinol. 13 (4), 399–404. 10.1677/joe.0.0130399 13345955

[B61] Wilson D. R. Thiel G. Arce M. L. Oken D. E. (1967). Glycerol induced hemoglobinuric acute renal failure in the rat. 3. Micropuncture study of the effects of mannitol and isotonic saline on individual nephron function. Nephron 4 (6), 337–355. 10.1159/000179594 6065553

[B62] Wilson M. E. Ford S. P. (2001). Comparative aspects of placental efficiency. Reprod. Suppl. 58, 223–232. 10.1530/biosciprocs.16.0016 11980192

[B63] Woods L. L. Ingelfinger J. R. Nyengaard J. R. Rasch R. (2001). Maternal protein restriction suppresses the newborn renin-angiotensin system and programs adult hypertension in rats. Pediatr. Res. 49 (4), 460–467. 10.1203/00006450-200104000-00005 11264427

[B64] Woods L. L. Weeks D. A. Rasch R. (2004). Programming of adult blood pressure by maternal protein restriction: role of nephrogenesis. Kidney Int. 65 (4), 1339–1348. 10.1111/j.1523-1755.2004.00511.x 15086473

[B65] Wysocki J. Ortiz-Melo D. I. Mattocks N. K. Xu K. Prescott J. Evora K. (2014). ACE2 deficiency increases NADPH-mediated oxidative stress in the kidney. Physiol. Rep. 2 (3), e00264. 10.1002/phy2.264 24760518 PMC4002244

[B66] Zou C. Wang C. Lu L. (2022). Advances in the study of subclinical AKI biomarkers. Front. Physiol. 13, 960059. 10.3389/fphys.2022.960059 36091391 PMC9449362

