# Supplementary figures and images for: Parental recovered acute kidney injury causes prenatal renal dysfunction and fetal growth restriction with sexually dimorphic implications for adult offspring
Source: Front Physiol. 2024 Apr 12;15:1357932. doi: 10.3389/fphys.2024.1357932 (PMC11045984; doi:10.3389/fphys.2024.1357932)

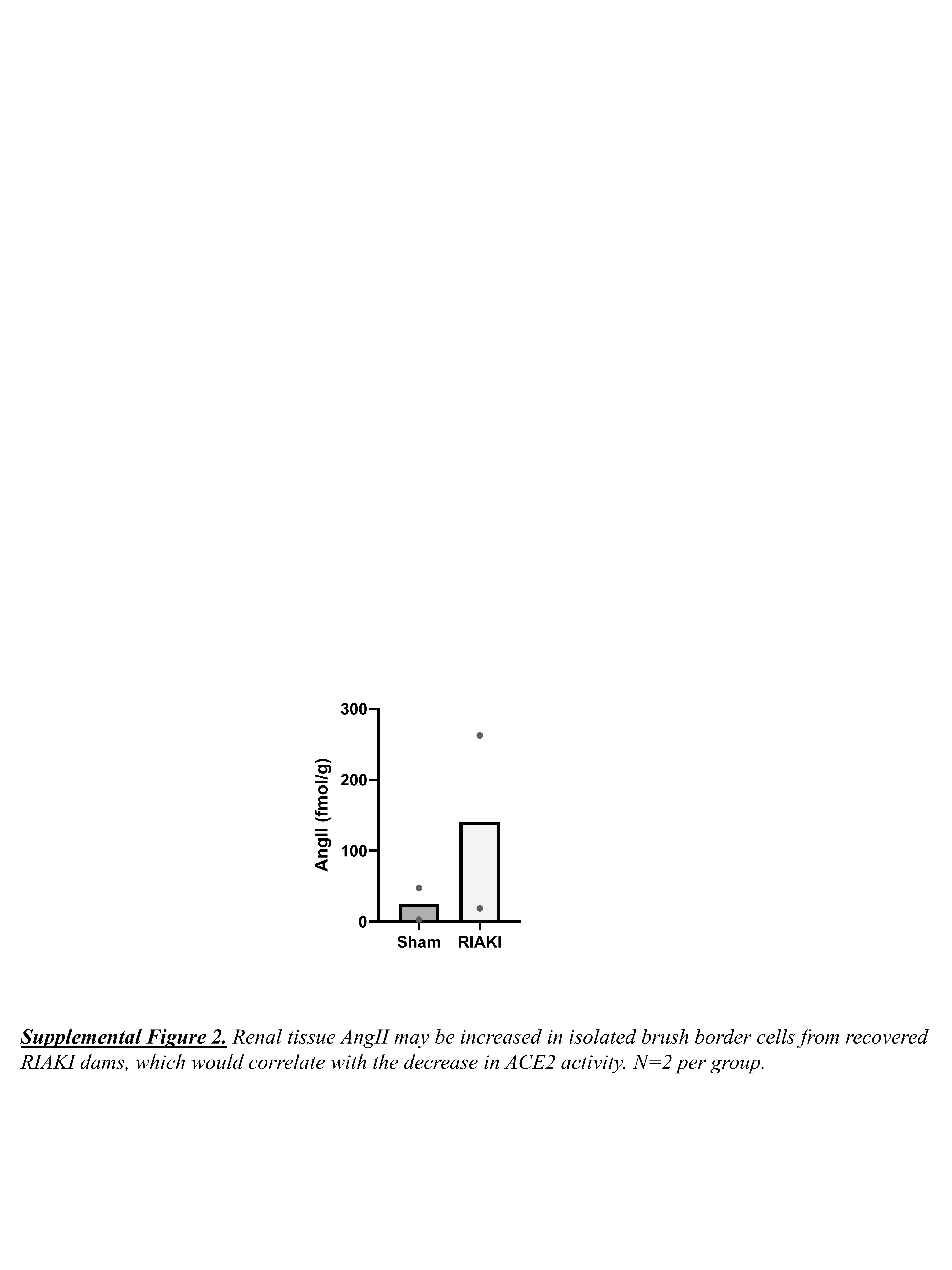

Supplement: Supplementary file 1 [file Image2.TIF]

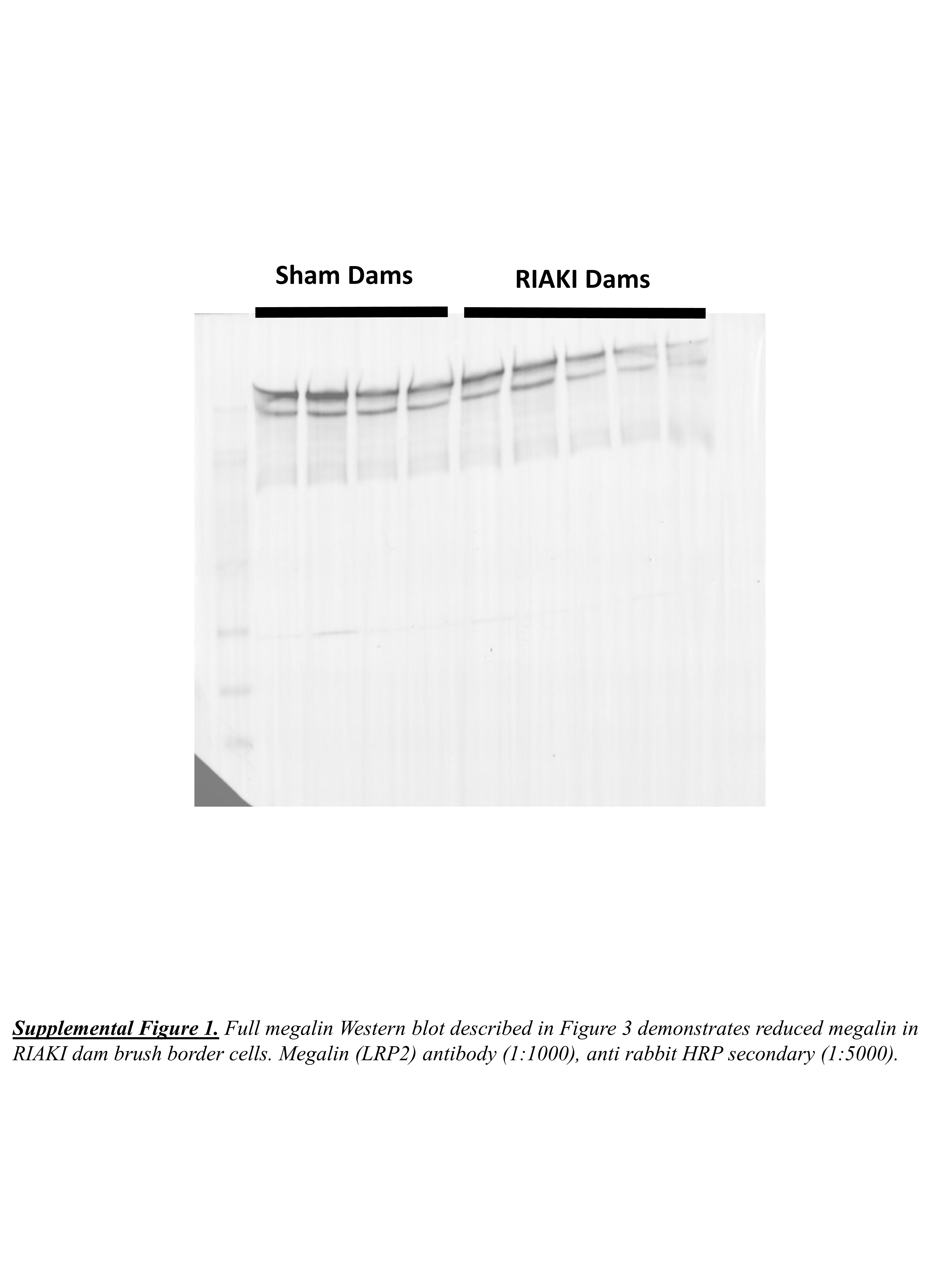

Supplement: Supplementary file 2 [file Image1.TIF]
